# Rational Design and Optimization of Novel PDE5 Inhibitors for Targeted Colorectal Cancer Therapy: An In Silico Approach

**DOI:** 10.3390/ijms26051937

**Published:** 2025-02-24

**Authors:** Samson Marvellous Oladeji, Deborah Ngozi Conteh, Lukman Abidemi Bello, Abayomi Emmanuel Adegboyega, Oluwatosin Sarah Shokunbi

**Affiliations:** 1 Department of Chemistry, Purdue University, West Lafayette, IN 47907, USA; soladeji@purdue.edu (S.M.O.); bellol@purdue.edu (L.A.B.); 2 Department of Medicine and Surgery, University of Ilorin, P.M.B, Ilorin 1515, Nigeria; debbieconteh@gmail.com; 3 Department of Biological Sciences, Structural and Computational Biology, Purdue University, West Lafayette, IN 47907, USA; abayomiadegboyega5@gmail.com; 4 Jaris Computational Biology Centre, Bioinformatics Unit, Jos 930241, Nigeria; 5 Department of Basic Sciences, Babcock University, School of Science and Technology, Babcock University, Ilishan-Remo 121003, Nigeria

**Keywords:** PDE5 inhibitors, exisulind, colorectal cancer, in silico design, ADMET, molecular dynamics

## Abstract

Colorectal cancer (CRC) is one of the leading causes of cancer-related deaths globally. Current treatment options including chemotherapy and targeted therapies face challenges such as resistance and toxicity. Cyclic guanosine monophosphate (cGMP)-specific phosphodiesterase 5 (PDE5) has emerged as a promising target for CRC therapy due to its role in regulating cellular processes like proliferation and apoptosis. This study focuses on the in silico design of a novel PDE5 inhibitor MS01 derived from the lead compound exisulind which has shown apoptotic effects but failed due to hepatotoxicity. Using Schrödinger’s Induced Fit Docking (IFD) and molecular dynamic simulations, MS01 was designed to enhance binding affinity and reduce toxicity. The docking studies showed that MS01 exhibits stronger interactions with key PDE5 residues, particularly Gln817 and Phe820. ADMET predictions indicate favorable pharmacokinetic profiles, with reduced risk of drug–drug interactions and improved bioavailability. Toxicity assessments revealed that MS01 and its analogs have moderate toxicity, with MS20 and MS21 demonstrating lower hepatotoxicity compared to exisulind. These findings suggest that MS01 has the potential to be a more effective and safer PDE5 inhibitor for CRC treatment pending further experimental validation.

## 1. Introduction

Colorectal cancer (CRC) ranks as the second leading cause of cancer-related deaths and the third most prevalent malignancy worldwide. In 2018, approximately 1.8 million new CRC cases were reported, resulting in 881,000 fatalities, representing nearly 10% of global cancer incidence and mortality [1]. The number of new cases is projected to rise to nearly 2.5 million by 2035 [2]. In the USA, the death rate from CRC decreased by approximately 50% in 2016 compared to 1970, due to advancements in screening and treatment methods. However, this reduction is largely observed in highly developed nations [2]. While the overall 5-year survival rate for CRC is approximately 64%, it drops significantly to 12% in metastatic cases, emphasizing the need for continued research to improve treatment outcomes [3].

Conventional therapies, such as surgery, chemotherapy, and radiation, have improved patient outcomes; however, these treatments are often associated with severe side effects and limited efficacy in advanced stages of the disease. Targeted therapy has emerged as an alternative treatment strategy that has effectively extended the overall survival of colorectal cancer (CRC) patients. Targeted therapies, such as anti-EGFR (epidermal growth factor receptor) or anti-VEGF (vascular endothelial growth factor) agents, can be used when specific molecular targets are identified, particularly in metastatic colon cancer [4]. Several targeted drugs have been developed and researched. These therapies act on cancer cells by directly inhibiting their proliferation, differentiation, and migration [4]. Targeted drugs can also modify the tumor microenvironment, including local blood vessels and immune cells, to hinder tumor growth and enhance immune surveillance and attack [5]. The main types of targeted drugs are monoclonal antibodies and small molecule inhibitors. Cetuximab became the first targeted therapy for colorectal cancer (CRC) to receive FDA approval in 2004, followed by bevacizumab later that year. Since then, several other targeted drugs for CRC have gained FDA approval, with ongoing development of new therapies. These drugs offer the advantage of being selected based on the molecular characteristics of tumor types, unlike traditional chemotherapy [6].

While targeted therapies have advanced CRC treatment, they are not without limitations, including resistance mechanisms and toxicity [7]. In recent years, attention has shifted to alternative molecular targets that may provide more selective and effective treatment options. One such promising target is cyclic nucleotide phosphodiesterases (PDEs), a family of enzymes that regulate intracellular signaling by degrading cyclic nucleotides such as cyclic adenosine monophosphate (cAMP) and cyclic guanosine monophosphate (cGMP) [8]. These molecules play crucial roles in cellular processes such as proliferation, differentiation, and apoptosis [9]. PDE inhibitors have shown the potential to influence these pathways selectively, based on the specific isoforms present in different tissues, thus minimizing side effects often seen with more conventional cancer therapies like EGFR and VEGF inhibitors [10]. This makes PDEs an attractive target in cancer therapy, particularly for colorectal cancer.

Among the PDEs, PDE5 has gained attention for its involvement in various physiological and pathological processes, including cancer [10,11]. PDE5 is a key regulator of cyclic guanosine monophosphate (cGMP) signaling, playing a crucial role in various physiological processes, including vascular tone, metabolism, renal function, and apoptosis [11]. The cGMP signaling pathway is initiated by guanylyl cyclase (GC) activation, leading to increased cGMP production, which exerts its effects through effectors like cGMP-dependent protein kinase G (PKG) and cGMP-regulated ion channels [10]. PDE5 is allosterically activated by cGMP binding to its regulatory domain, enhancing its catalytic activity to hydrolyze cGMP into inactive 5′-GMP, thereby restoring basal cGMP levels. Expressed in multiple tissues such as the lung, brain, heart, and vascular smooth muscle, PDE5 function is also modulated by genetic expression, phosphorylation (activation), and nitrosylation (degradation) [8]. Structurally, PDE5 contains a C-terminal catalytic domain and N-terminal regulatory GAF (mammalian cGMP-dependent phosphodiesterase, Anabaena adenylyl cyclase, and Escherichia coli FhlA) domains, with the GAFa domain binding cGMP to regulate activity and the GAFb domain facilitating enzyme dimerization [12]. This regulation ensures precise control of cGMP-dependent cellular processes, particularly in response to nitric oxide-activated soluble GC signaling.

PDE5 inhibitors, like sildenafil, have demonstrated potential anti-tumor effects in preclinical studies, suggesting that targeting PDE5 may be a promising strategy for cancer therapy [13,14,15]. In this paper, we aim to develop a potent selective PDE5 inhibitor for the treatment of colorectal cancer. Modifications were made to a previously reported small molecule PDE5 inhibitor, exisulind, the lead compound in a series of selective apoptotic antineoplastic drugs (SAANDs) developed by OSI Pharmaceuticals [16,17]. These modifications aim to enhance the efficacy and improve the toxicity profile of the new lead compound. In silico molecular docking studies using Schrodinger’s Induced Fit Docking (IFD) workflow demonstrate that the proposed novel compounds could be a more potent inhibitor of PDE5 compared to the reported lead compounds, based on binding energy, interactions, and drug-likeness.

## 2. Results and Discussion

### 2.1. Lead Compound Development

**Figure 1:**
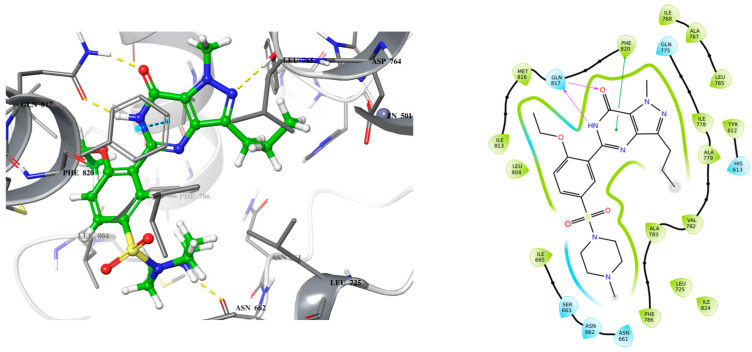
3D (**left**) and 2D (**right**) crystal structure of sildenafil bound to PDE5 (PDB ID: 2H42).

**Figure 2:**
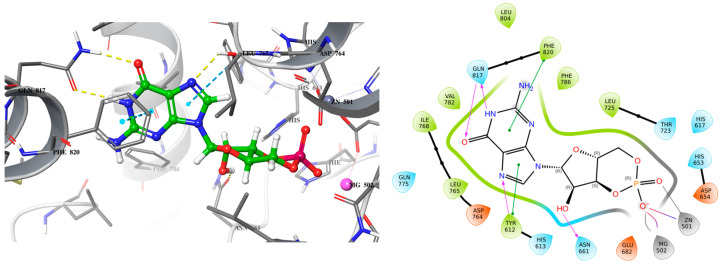
3D (**left**) and 2D (**right**) docking pose of cGMP bound to PDE5 (PDB ID: 2H42).

**Figure 3:**
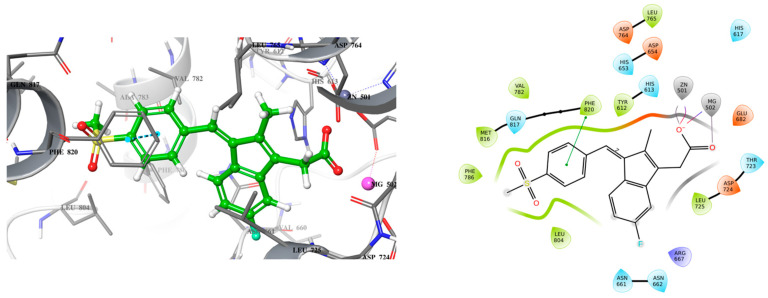
3D (**left**) and 2D (**right**) docking pose of exisulind bound to PDE5 (PDB ID: 2H42).

Exisulind is known to have the ability to induce apoptosis in many cancer cell lines including colorectal cancer through cGMP PDE5 inhibition [17,18]. However, exisulind failed to gain FDA approval primarily due to significant safety concerns (hepatotoxicity) [19]. This failure underscores the need for optimization in the development of safer and more potent analogs. Despite its promise, no crystal structure of PDE5A bound with exisulind is available, which poses a challenge for structure-based drug design. Given that sildenafil, an FDA-approved PDE5 inhibitor, has demonstrated similar apoptotic effects in some cancer cell lines, including colorectal cancer cells, it provides a useful model for lead optimization [13,14,15]. Sildenafil’s crystal structure in complex with PDE5 offers insights into the binding interactions necessary for effective inhibition of the enzyme (Figure 1). To begin the design of a new lead compound, the docking procedure was validated by using Schrödinger’s Induced Fit Docking (IFD) to dock sildenafil with PDE5, as outlined in the Section 3. After successful validation of the docking protocol, cyclic GMP (the native substrate of PDE5) and exisulind were also docked to PDE5 (Figure 2 and Figure 3). This dual approach helps us build on the known interactions of sildenafil and exisulind with PDE5, aiming to create a novel compound that retains the apoptosis-inducing effects of exisulind while addressing the safety concerns that led to its regulatory failure.

Analyzing the crystal structures and docking results reveals significant interactions involving the Gln817 residue, which acts as both an H-bond donor and acceptor with the pyrazolopyrimidinone group of sildenafil and guanine of cGMP. Additionally, there is a hydrophobic interaction between the Phe820 residue and the purine in cGMP, as well as with pyrimidinone in sildenafil. Another key interaction is metal binding, observed with the phosphate group in cGMP and the carboxylic acid in exisulind. The literature has shown that Gln817 is crucial for nucleotide selectivity in PDE5, while the purine base is held tightly in the active site by a “hydrophobic clamp” formed by Phe820 [20,21].

These structural insights highlight the importance of specific molecular interactions in drug efficacy and selectivity, which directly inform our approach to addressing exisulind’s safety concern. The cause of exisulind’s hepatotoxicity is not well understood, although it has been proposed that the toxicity may be due to a reactive metabolite formed by the drug [16]. Additionally, exisulind contains a carboxylic acid group, which has been associated with drug-induced liver injury at higher concentrations [22]. Our approach is to retain the carboxylic group first, as it plays a crucial role in interacting with metal ions, while improving the drug’s potency by enhancing its binding affinity for PDE5. Increasing the potency should allow for a reduced dosage, potentially decreasing the associated toxicity. Exisulind is a weak inhibitor of PDE5, with an IC_50_ of 128 µM, but it is known to inhibit PDE5 sufficiently to sustain elevated cGMP levels in colon cancer cells, thereby triggering apoptosis [17].

**Figure 4:**
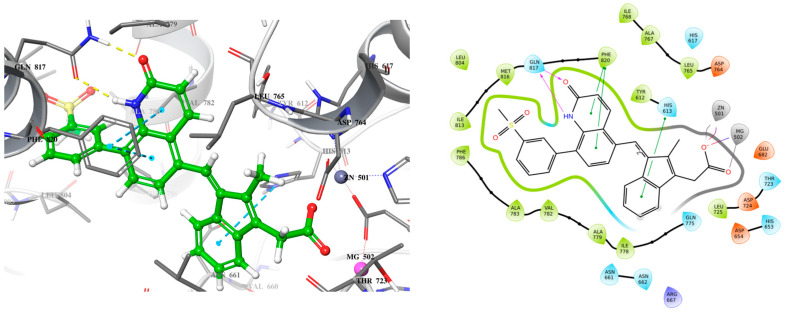
3D (**left**) and 2D (**right**) docking pose of MS01 bound to PDE5.

Building on these findings, we sought to design a more effective inhibitor that engages crucial residues like Gln817, which is vital for PDE5 catalysis. To achieve this, we replaced the sulfonyl benzene ring of exisulind with a 2-quinolone scaffold in MS01 (Figure 4). Quinolones are versatile scaffolds in medicinal chemistry due to their structural flexibility, binding affinity, and synthetic accessibility allowing for diverse chemical modifications that can target various biological pathways. Their planar structure and favorable pharmacokinetic properties, including good bioavailability, make it an excellent core for developing potent and effective drugs [23]. In MS01, the 2-quinolone mimics the H-bond donor and acceptor features seen in the pyrazolopyrimidinone group of sildenafil and guanine in cGMP, enabling it to form a bidentate H-bond with Gln817 residue. Additionally, MS01 preserves the hydrophobic clamp formed by Phe820, which stabilizes PDE5 substrates in the active site. The IFD score in Table 1 showed that MS01 binds more strongly with PDE5 than exisulind and cGMP. The IFD score evaluates how well a ligand fits into a receptor’s binding site, considering both the ligand’s shape and the receptor’s flexibility.

### 2.2. Molecular Dynamic Simulation

**Figure 5:**
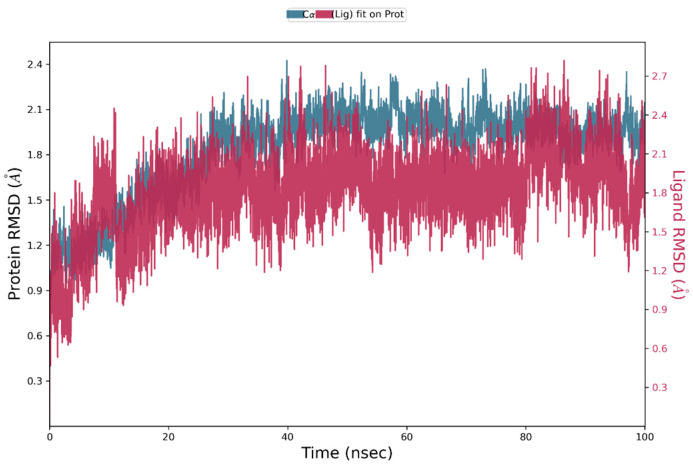
RMSD plot of PDE5-MS01 complex during 100 ns simulations.

In order to know whether the MS01-PDE5 complex is stable, MD simulation was performed using the best MS01-PDE5A pose from IFD docking. Figure 5 presents the root-mean-square deviation (RMSD) profiles of PDE5A and MS01. The protein exhibits a steady fluctuation in RMSD, ranging from 1 to 2.1 Å for the first 23 ns, followed by more stable interactions for the rest of the simulation, indicating overall stability throughout the simulation. MS01, on the other hand, fluctuates with maximum 2.1 Å for the first 15 ns, followed by more stable interactions for the rest of the simulation. The red spectrum in the plot illustrates the RMSD of the ligand, where the protein–ligand complex is first aligned using the protein backbone of the reference structure, followed by measuring the RMSD of the ligand’s heavy atoms.

**Figure 6:**
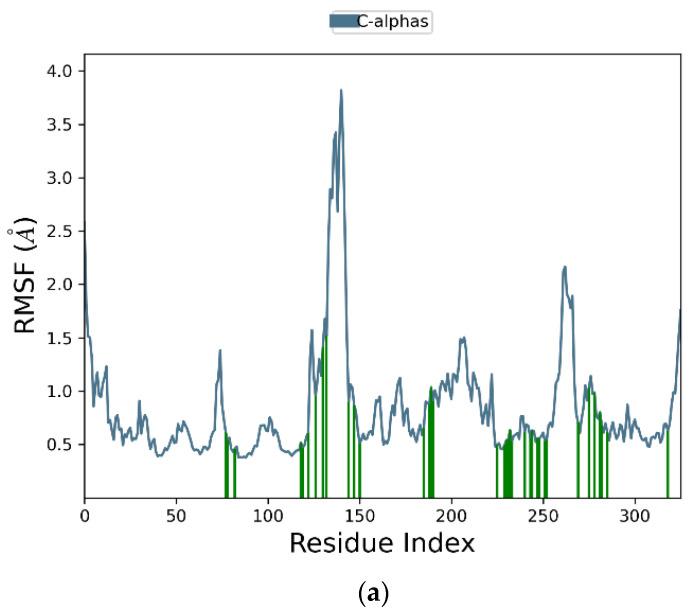

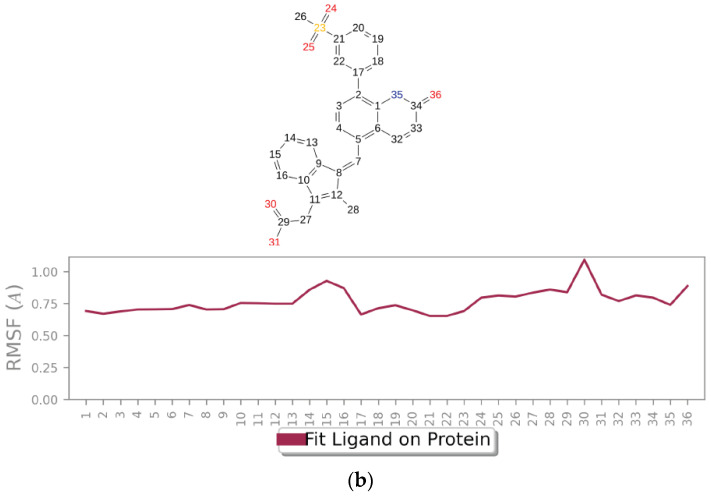
Time-evolving root-mean-square fluctuation (RMSF) of (**a**) the protein residues (**top**) and (**b**) the ligand (**bottom**).

Figure 6a graphically represents the root-mean-square fluctuation (RMSF) values for each residue in the protein backbone. In these plots, peaks correspond to residues exhibiting the highest fluctuations throughout the simulation. The fluctuations range from 2.5 Å to 3.7 Å. The maximum fluctuation was observed between residues 120 and 148, indicating regions of higher flexibility, while residues 250 to 270 also show significant fluctuations. In contrast, the rest of the residues showed less fluctuation during the entire 100 ns simulation. Protein residues interacting with the ligand are indicated by green vertical bars. Figure 6b presents the ligand RMSF plot, depicting atomic-level fluctuations. The ligand exhibits minimal fluctuations at 0.65 Å and maximum fluctuations at 1.1 Å, indicating stable conformational behavior throughout the simulation.

**Figure 7:**
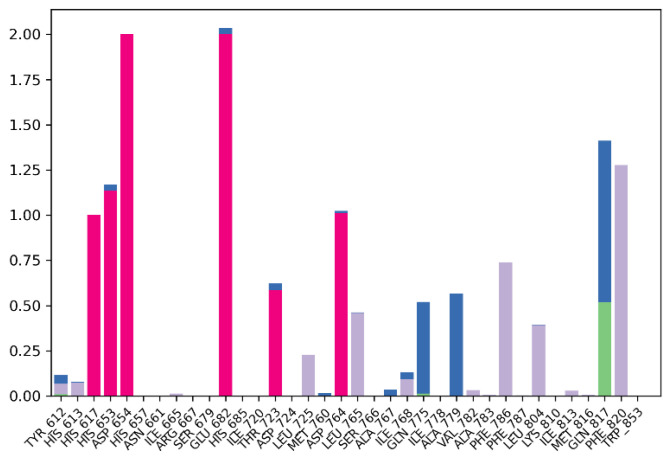
Protein–ligand contact bar diagram of PDE5-MS01 complex during 100 ns simulations.

Protein–ligand interactions are categorized into four types: hydrogen bonds, hydrophobic, ionic, and water bridges. Simulation results in Figure 7 indicate that the protein–ligand complex exhibited mostly ionic interaction between the residues (red). The stacked bar charts are normalized over the course of the trajectory: for example, a value of 0.7 suggests that 70% of the simulation time the specific interaction is maintained. Values over 1.0 are possible, as some protein residue may make multiple contacts of same subtype with the ligand. The residues Glu682 (2.00) and Asp654 (2.00) exhibited the most ionic interaction throughout the simulation period, followed by His653 (1.15) and Asp764 (1.10). In addition, the residue Phe820 (1.25) exhibited the most hydrophobic bonding (gray) throughout the simulation period, followed by Phe786 (0.70) and Leu765 (0.45). Only residue Gln817 exhibited hydrogen bonding (green) up to 50% of the simulation time in addition to the water bridge (blue) that it exhibited. Overall, the simulation indicates presence of ionic bonds, hydrophobic interactions, hydrogen bonds, and water bridges throughout the simulation period. This indicates stability of the complex.

### 2.3. Lead Compound Optimization

After the development of the new lead compound, different structural modifications were designed to investigate PDE5A inhibition and improve pharmacokinetic properties. The modifications targeted two segments of MS01: the first modifications were focused on the quinolone binding moiety, and the second involved changing of substituent groups, nitrogen scan, and introduction of groups that help increase drug-like properties and binding affinity.

#### 2.3.1. Changing the Quinolone Scaffold

**Figure 8:**
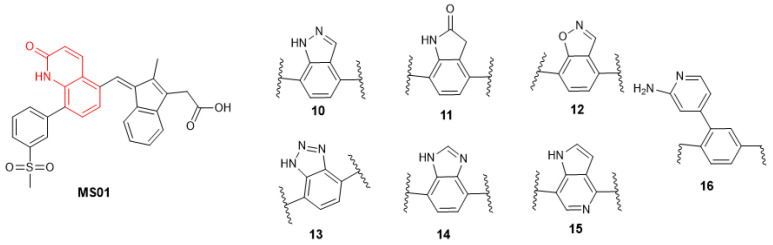
Lead compound structural modification, part 1.

The quinolone binding site features both hydrogen bond donor and acceptor which make important interactions with the Gln817 residue of the active site. Many privileged scaffolds exhibit similar binding characteristics to quinolone and are present in several FDA-approved drugs [23,24]. We proposed to test seven additional privilege scaffolds (MS10–MS16), as shown in Figure 8.

#### 2.3.2. Other Changes to Improve Drug-like Properties

**Figure 9:**
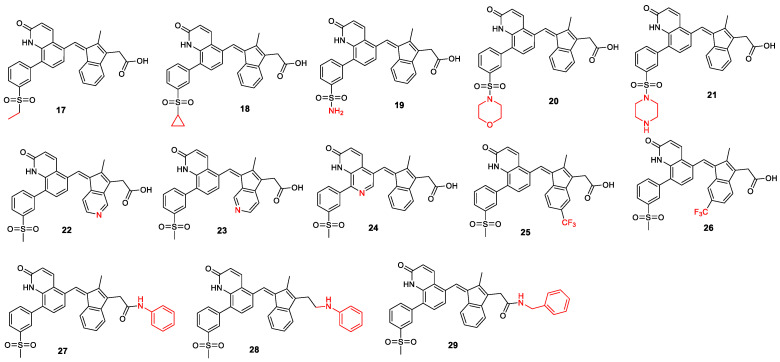
Lead compound structural modification, part 2.

The second set of analogs focused on modifying the substituents to improve drug-like properties (Figure 9, the changes to the lead compound are marked in red). We began with a nitrogen scan by systematically replacing one of the carbons in the ring system with nitrogen, one at a time, and scanning through the ring [22,23,24]. The literature indicates that such modifications can have significant effects on lead compound optimization [25,26]. Additionally, modifications were made to the solvent-exposed methyl sulfonyl group. Analogs containing acyl groups, sulfonyl cyclopropane, sulfonamide, sulfone morpholine, and sulfone piperazine were designed, as these are important substituents used to tune drug pharmacokinetics and, in some cases, activity in medicinal chemistry (MS17–MS21). We also designed some trifluoromethyl analogs (MS25 and MS26), as the trifluoromethyl group is highly valued in medicinal chemistry for its ability to enhance metabolic stability and lipophilicity, often leading to improved pharmacokinetics [27]. Finally, amide and amine analogs (MS27–MS29) were designed. The literature examples, such as CP461, CP248, SSA, and SBA, have shown that amide and amine analogs of exisulind exhibit improved IC_50_ against PDE5A and EC_50_ against colon cancer cell lines [17,28,29]. Since the carboxylic acid group is associated with drug-induced liver injury (DILI), these analogs have the potential to improve the drug’s safety profile.

Table 2 presents the docking scores for MS01 and its analogs, alongside controls (exisulind, sildenafil, and the native PDE5 substrate cGMP). Compared to the controls, compounds MS25, MS18, MS23, and MS01 (lead) exhibit stronger binding affinities for PDE5, suggesting that they are potentially more effective inhibitors. This preliminary analysis indicates that these novel analogs show promise in their ability to inhibit PDE5.

### 2.4. ADMET Property Prediction

ADMET (Absorption, Distribution, Metabolism, Excretion, and Toxicity) refers to the pharmacokinetic and toxicological properties of a drug candidate, determining its efficacy and safety in biological systems. Table 3 summarizes the predicted lipophilicity, drug-likeness, and bioavailability scores of the compounds, as assessed using SwissADME. Lipophilicity, indicated by Log *p* values, plays a critical role in determining a compound’s ability to permeate biological membranes. A Log *p* value between 0 and 5 is generally considered ideal, balancing solubility and permeability [30]. MS01 and analogs have Log *p* values below 5, except for trifluoromethane analogs (MS25 and MS26) and amide and amine analogs (MS26 to MS29). Solubility, as determined by the ESOL method, offers valuable insight into a compound’s ability to dissolve in aqueous environments, such as the gastrointestinal tract. The ESOL model estimates a compound’s aqueous solubility directly from its molecular structure [31]. The Log S values for the compounds range from −3.67 to −7.12. Based on the solubility scale, most of the compounds are moderately soluble (Log S < −4 and >−6), with MS21 being the most soluble (Log *p* −3.67) and MS28 being the least soluble (Log *p* −7.12). Another critical parameter is the topological polar surface area (TPSA), which is directly related to a molecule’s ability to permeate cellular membranes [32]. The TPSA values for the compounds, ranging from 105.85 to 127.95 Å^2^, are below the 140 Å^2^ threshold typically associated with good membrane permeability. This suggests that these compounds are likely to effectively cross cell membranes, which is crucial for intracellular drug targets like phosphodiesterases. Additionally, nine of the compounds violate Lipinski’s Rule of Five. However, only MS16 violates Verber’s rule. These rules assess properties like molecular weight, lipophilicity, bioavailability, and hydrogen bond donors/acceptors, and adherence to these guidelines is a positive indication of good absorption and permeability [33]. Interestingly, the lead compound MS01 and one of the most potent analogs according to binding energies, MS13, adhere to both rules. For bioavailability predictions, all compounds, except MS27 to MS29, exhibit a score of approximately 0.56, suggesting a minimum of 10% oral bioavailability in rats or detectable permeability in human colon carcinoma (Caco-2) cells. In contrast, MS27 to MS29 have a lower score of 0.17, suggesting only a 17% probability of achieving similar bioavailability, based on the model proposed by Testa and Kraemer [34].

Table 4 presents the results of the pharmacokinetic profile of MS01 and its analogs. One of the primary considerations is gastrointestinal (GI) absorption, where all compounds, including MS01, exhibit low absorption rates. This indicates that if these compounds are intended for oral administration, strategies to improve their bioavailability, such as formulation adjustments, might be necessary [33]. However, if alternative administration routes (such as intravenous) are pursued, this may not be a significant limitation. Another important feature is the lack of blood–brain barrier (BBB) permeability observed in all the tested compounds [31,35]. This characteristic is advantageous for drugs that are intended to target peripheral tissues, as it minimizes the risk of central nervous system side effects. Given that MS01 and its analogs are being considered for use in colorectal cancer treatment, the absence of BBB permeability might not be a major problem. The analysis also reveals that none of the compounds, except MS21, are substrates for P-glycoprotein (Pgp). Pgp is an efflux transporter that can reduce the intracellular concentration of drugs by pumping them out of cells [36]. Being a non-substrate means that these compounds are less likely to be actively expelled from cells, enhancing their potential effectiveness, particularly in cancer cells where Pgp is often overexpressed. This property is especially favorable for MS01 and its analogs, as it may increase their intracellular retention and potency. Regarding cytochrome P450 (CYP) enzyme inhibition, most of the compounds demonstrate inhibitory effects on CYP2C19 and CYP2C9 but not on CYP1A2, CYP2D6, and CYP3A4. Interestingly MS16, MS27, and MS28 are predicted to have no inhibitory effect on all the tested cytochrome isoforms. Cytochrome P450 (CYP) enzymes form a large superfamily of isoenzymes that play a crucial role in phase I drug metabolism. Inhibiting major isoforms such as CYP1A2, CYP3A4, CYP2C9, CYP2C19, and CYP2D6 can lead to pharmacokinetic drug interactions, as these enzymes are frequently involved in drug metabolism [31,37]. Evaluating the extent to which compounds inhibit these enzymes is essential for determining their potential to cause drug interactions and for improving their pharmacokinetic properties.

The lack of CYP3A4 inhibition shown by most of the compounds is a positive finding, as CYP3A4 is one of the most common enzymes involved in drug metabolism [38]. This reduces the likelihood of adverse interactions with a broad range of medications. However, the inhibition of CYP2C19 and CYP2C9 suggests that careful monitoring and consideration of potential interactions with drugs metabolized by these enzymes will be necessary in future development stages. The table also presents the skin permeation values (log Kp in cm/s) for the test compounds, ranging from −9.41, indicating low permeability, to −5.46, representing high permeability. MS21 exhibits the lowest skin permeability, while MS28 shows the highest. The range of values for each compound suggests that all tested compounds possess some level of permeability [31]. This suggests that transdermal delivery is likely to be an effective route for these compounds.

Table 5 provides ADMET data outlines for toxicity profiles of MS01 and its analogs, focusing on various parameters such as LD50 values (in mg/kg), toxicity class, and specific toxicological endpoints like hepatotoxicity, respiratory toxicity, carcinogenicity, immunotoxicity, mutagenicity, and cytotoxicity. All the compounds, including MS01, fall into toxicity classes 3 to 6, which correspond to moderate toxicity, with LD50 values ranging between 264 to 20,000 mg/kg. Class 3 is toxic if swallowed (50 < LD50 ≤ 300), and 6 is non-toxic (LD50 > 5000). Conversely, most of the compounds are moderately toxic, while MS11 and MS27 are considered non-toxic [39]. Furthermore, MS01, along with its analogs, shows consistent hepatotoxicity and respiratory toxicity, while being inactive in terms of carcinogenicity, immunotoxicity, mutagenicity, and cytotoxicity. Interestingly, MS20 and MS21 are predicted to be inactive in all the toxicity tests, including hepatoxicity, except for respiratory toxicity. Overall, this suggests that while the compounds pose certain risks to liver and lung health, their overall mutagenic and carcinogenic potential remains low. The hepatotoxicity observed in all the compounds except for MS20, MS21, MS28, and MS29 may relate to the metabolic processing of the compounds in the liver, a known challenge for drugs like exisulind, which is structurally related to MS01. Exisulind’s hepatotoxic effects have been linked to the formation of reactive intermediates during metabolism, which can cause liver damage [16,19]. The active respiratory toxicity might also be a consideration for drug safety, potentially reflecting the compounds’ interaction with key enzymes or signaling pathways within respiratory tissues.

## 3. Materials and Methods

### 3.1. Ligand Preparation

The LigPrep module from the Schrödinger Suite (version 2020-3) was employed for preparing all the ligands used in the molecular docking studies. The OPLS4 force field was applied to model the interactions between atoms within the system. The ionization states of each ligand at a physiological pH of 7.2 ± 0.2 were calculated, ensuring the correct low-energy 3D structures and stereochemistry. The stereoisomers of the ligands were generated while preserving the original chirality.

### 3.2. Protein Preparation

The crystal structure of cGMP phosphodiesterase 5 (PDB ID: 2H42) was obtained from the Protein Data Bank (PDB). Protein preparation was carried out using the Protein Preparation Wizard in Glide (Schrödinger Suite 2020-3), where hydrogen atoms were added, bond orders were assigned, and disulfide bonds were formed. Missing loops and side chains were completed using Prime 3.3. Water molecules located more than 3.0 Å from heteroatoms were removed, and the structure was subsequently minimized using the OPLS4 force field and optimized with PROPKA to refine protonation states [40]. Following this, a receptor grid file was generated to define the ligand binding site.

### 3.3. Receptor Grid Generation

To define the size and location of the protein’s active site for ligand docking, the receptor grid generation tool in Schrödinger Maestro 12.5 was used. The scoring grid was based on the co-crystallized ligand (PDE5 in complex with sildenafil). A van der Waals (vdW) radius scaling factor of 1.0 with a partial cutoff of 0.25 was applied to nonpolar receptor atoms during the grid generation process.

### 3.4. Molecular Docking

Molecular docking simulations were performed by generating a receptor grid file using the Glide tool within Schrödinger Maestro 12.5. Standard precision (SP) and flexible ligand sampling were utilized as docking parameters. A 0.15 partial charge cutoff was applied to ligand atoms, and a vdW radius scaling factor of 0.80 was used for the docking process.

### 3.5. Induced Fit Docking (IFD)

The interaction between PDE5A and the ligands exisulind, sildenafil, cGMP, and MS01 was examined using the Induced Fit Docking (IFD) tool in Schrödinger Maestro 12.5. This method assumes significant receptor conformational changes upon ligand binding and optimizes ligand positioning accordingly. Initially, the receptor structure was minimized under constraints before docking the ligands using softened potentials in Glide. The resulting docked poses were grouped into 20 clusters and passed to Prime for further refinement. Prime was used for side-chain prediction and minimization, after which the optimized receptor structures were redocked using Glide. The system automatically determined solvent-accessible surface areas, salt bridges, B-factors, rotamer searches, and van der Waals scaling factors. The first stage of docking produced a large number of poses, which were filtered to retain up to 80 poses per ligand for further evaluation. Final docking scores were calculated using the Glide SP module.

### 3.6. Molecular Dynamic Simulation

Following molecular docking, molecular dynamic simulations were conducted for the protein–ligand complexes using Desmond 2.0, a software package from Schrödinger LLC, New York, NY, USA. The simulations were run for 100 nanoseconds. System preparation was conducted using the ‘System Builder’ tool in Maestro, with TIP3P (Transferable Intermolecular Potential with 3 Points) as the solvent model in an orthorhombic box (10 × 10 × 10 Å). Counter ions (NaCl, 3.367 mM) were added to neutralize the system. The OPLS_2005 force field and RESPA integrator parameters were applied during the simulation. All systems were relaxed before the simulations, and the NPT ensemble was used, maintaining a temperature of 310 K and a pressure of 1 atm. Trajectories were recorded every 100 ps for further analysis, and the stability of the simulations was evaluated by monitoring the root-mean-square deviation (RMSD) of both the protein and ligand over time.

### 3.7. Pharmacology Parameters

The pharmacokinetic and toxicological properties (ADMET) of the test compounds were evaluated using integrated predictive models available on the SwissADME and PROTOX-3 servers. ADMET analysis encompasses absorption, distribution, metabolism, and toxicity predictions for the ligands [31,39].

## 4. Conclusions

This study presents the development of a novel selective PDE5 inhibitor, MS01, through in silico rational drug design aimed at improving colorectal cancer (CRC) treatment. Despite advancements in conventional therapies and targeted drugs, challenges such as resistance mechanisms, toxicity, and limited efficacy in advanced stages persist, highlighting the need for more effective interventions. PDE5, a key regulator of intracellular cyclic nucleotide signaling, has emerged as a promising molecular target for cancer therapy due to its role in regulating proliferation, differentiation, and apoptosis. Existing PDE5 inhibitors, such as sildenafil, have demonstrated potential anti-tumor effects, supporting PDE5’s viability as a target for CRC treatment. This work builds on exisulind, a cGMP PDE5 inhibitor with apoptotic effects, by modifying its structure to address its major limitation—hepatotoxicity. Through the Schrödinger Induced Fit Docking (IFD) workflow and molecular dynamic simulations, the newly designed MS01 compound was found to exhibit stronger binding interactions with key PDE5 residues, particularly Gln817 and Phe820, which are vital for substrate stabilization and enzymatic activity. Molecular dynamic simulations confirmed the stability of the MS01-PDE5 complex with favorable root-mean-square deviation (RMSD) and fluctuation (RMSF) profiles over 100 nanoseconds. Additionally, ADMET (Absorption, Distribution, Metabolism, Excretion, and Toxicity) predictions indicated that MS01 and its analogs possess drug-like properties, including suitable lipophilicity, solubility, and bioavailability. While some analogs violated Lipinski’s Rule of Five, most adhered to drug-likeness guidelines and demonstrated promising pharmacokinetic profiles, including the absence of blood–brain barrier (BBB) permeability—traits favorable for targeting peripheral tissues like the colon. Moreover, minimal CYP3A4 inhibition reduces the likelihood of drug–drug interactions, though inhibitory effects on CYP2C19 and CYP2C9 require further attention. Toxicity assessments placed most compounds in toxicity classes 3 to 6, indicating moderate toxicity with some analogs, particularly MS20 and MS21, showing reduced hepatotoxicity and other toxicological risks. Overall, MS01 and its analogs offer promising prospects as PDE5 inhibitors for CRC therapy. However, experimental validation, toxicity refinement, and pharmacokinetic optimization remain essential for clinical translation.

## Figures and Tables

**Table 1:** Induced Fixed Docking scores of sildenafil, exisulind, cGMP, and MS01 bound to PDE5A (PDB: 2H42).

Compounds	IFD Score
Sildenafil	−765.04
Exisulind	−756.8
cGMP	−756.22
MS01	−761.16

**Table 2:** In silico docking scores of MS01, MS01 analogs, exisulind, sildenafil, and cGMP using Glide docking (SP) protocol in Schrödinger.

Compound ID	ΔG Energy (Kcal/mol)
MS25	−14.729
MS18	−14.441
MS23	−13.984
MS01-Lead	−13.798
MS21	−13.092
MS16	−13.073
MS17	−12.772
MS20	−12.666
MS26	−12.618
MS22	−12.560
MS24	−12.082
MS12	−11.992
MS19	−11.913
MS15	−11.892
MS11	−11.608
MS29	−11.164
MS10	−11.060
MS13	−10.850
MS14	−10.763
Sildenafil	−10.383
Exisulind	−9.895
cGMP	−9.798
MS28	−8.749
MS27	−7.952

**Table 3:** Predicted lipophilicity (Log *p*), water solubility (Log Sw), drug-likeness, and bioavailability scores.

Molecule	MW (g/mol)	Consensus Log *p*	Log S (ESOL)	TPSA	Fraction Csp3	Verber #Violations	Lipinski #Violations	Bioavailability Score
MS01	497.56	4.62	−5.26	112.68	0.1	0	0	0.56
MS10	470.54	4.35	−5.23	108.5	0.11	0	0	0.56
MS11	485.55	4.04	−4.76	108.92	0.14	0	0	0.56
MS12	471.52	4.57	−5.34	105.85	0.11	0	0	0.56
MS13	471.53	3.98	−4.96	121.39	0.12	0	0	0.56
MS14	470.54	4.31	−5.20	108.50	0.11	0	0	0.55
MS15	469.55	4.83	−5.54	95.61	0.11	0	0	0.56
MS16	522.61	4.86	−5.87	118.73	0.10	1	0	0.56
MS17	511.59	4.96	−5.5	112.68	0.13	0	2	0.56
MS18	523.6	5.05	−5.68	112.68	0.16	0	2	0.56
MS19	498.55	3.81	−4.84	138.7	0.07	0	0	0.56
MS20	568.64	4.24	−5.29	125.15	0.19	0	1	0.56
MS21	567.65	3.41	−3.67	127.95	0.19	0	1	0.55
MS22	498.55	3.82	−4.59	125.57	0.11	0	0	0.56
MS23	498.55	3.9	−4.59	125.57	0.11	0	0	0.56
MS24	498.55	3.96	−4.62	125.57	0.11	0	0	0.56
MS25	565.56	5.65	−6.13	112.68	0.13	0	2	0.56
MS26	565.56	5.62	−6.13	112.68	0.13	0	2	0.56
MS27	572.67	5.75	−6.46	104.48	0.09	0	2	0.17
MS28	558.69	6.28	−7.12	87.41	0.11	0	2	0.17
MS29	586.7	5.79	−6.43	104.48	0.11	0	2	0.17

**Table 4:** Pharmacokinetic prediction results for the test compounds.

Molecule	GI Absorption	BBB Permeant	Pgp Substrate	CYP1A2 Inhibitor	CYP2C19 Inhibitor	CYP2C9 Inhibitor	CYP2D6 Inhibitor	CYP3A4 Inhibitor	Log Kp (cm/s)
MS01	Low	No	No	No	Yes	Yes	No	No	−6.84
MS10	Low	No	No	No	Yes	Yes	No	No	−6.52
MS11	Low	No	No	Yes	Yes	Yes	No	No	−7.17
MS12	Low	No	No	No	Yes	Yes	No	No	−6.41
MS13	Low	No	No	No	Yes	No	No	No	−6.85
MS14	Low	No	No	No	Yes	Yes	No	No	−6.56
MS15	Low	No	No	No	Yes	Yes	No	No	−6.16
MS16	Low	No	No	No	No	No	No	No	−6.43
MS17	Low	No	No	Yes	Yes	Yes	No	No	−6.67
MS18	Low	No	No	Yes	No	Yes	No	No	−6.6
MS19	Low	No	No	No	Yes	No	No	No	−7.32
MS20	Low	No	No	No	No	Yes	No	No	−7.6
MS21	Low	No	Yes	No	No	Yes	No	No	−9.41
MS22	Low	No	No	No	Yes	Yes	No	No	−7.61
MS23	Low	No	No	No	Yes	Yes	No	No	−7.61
MS24	Low	No	No	No	Yes	Yes	No	No	−7.58
MS25	Low	No	No	Yes	No	No	No	No	−6.63
MS26	Low	No	No	Yes	No	No	No	No	−6.63
MS27	Low	No	No	No	No	No	No	No	−6.37
MS28	Low	No	No	No	No	No	No	No	−5.46
MS29	Low	No	No	Yes	No	No	No	Yes	−6.5

**Table 5:** Toxicity profiles of MS01 and analogs.

Molecule	LD50 mg/kg	Toxicity Class	Hepatotoxicity	Respiratory Toxicity	Carcinogenicity	Immunotoxicity	Mutagenicity	Cytotoxicity
MS01	800	4	Active	Active	Inactive	Inactive	Inactive	Inactive
MS10	1000	4	Active	Active	Inactive	Inactive	Inactive	Inactive
MS11	5000	5	Active	Active	Inactive	Inactive	Inactive	Inactive
MS12	1000	4	Active	Active	Inactive	Inactive	Inactive	Inactive
MS13	1000	4	Active	Active	Inactive	Inactive	Inactive	Inactive
MS14	264	3	Active	Active	Inactive	Inactive	Inactive	Inactive
MS15	264	3	Active	Active	Inactive	Inactive	Inactive	Inactive
MS16	264	3	Active	Active	Inactive	Inactive	Inactive	Inactive
MS17	800	4	Active	Active	Inactive	Active	Inactive	Inactive
MS18	800	4	Active	Active	Inactive	Inactive	Inactive	Inactive
MS19	800	4	Active	Active	Inactive	Inactive	Inactive	Inactive
MS20	300	3	Inactive	Active	Inactive	Inactive	Inactive	Inactive
MS21	300	3	Inactive	Active	Inactive	Inactive	Inactive	Inactive
MS22	264	3	Active	Active	Inactive	Active	Inactive	Inactive
MS23	264	3	Active	Active	Inactive	Active	Inactive	Inactive
MS24	800	4	Active	Active	Inactive	Inactive	Inactive	Inactive
MS25	264	3	Active	Active	Inactive	Active	Inactive	Inactive
MS26	264	3	Active	Active	Inactive	Active	Inactive	Inactive
MS27	20,000	6	Active	Active	Inactive	Inactive	Inactive	Inactive
MS28	264	3	Inactive	Active	Inactive	Active	Inactive	Inactive
MS29	200	3	Inactive	Active	Inactive	Active	Inactive	Inactive

## Data Availability

The data presented in this study are available on request from the corresponding author.
